# Prophylactic penehyclidine inhalation for prevention of postoperative pulmonary complications in high-risk patients: study protocol of a randomized controlled trial

**DOI:** 10.1186/s13063-017-2315-7

**Published:** 2017-11-28

**Authors:** Ting Yan, Xin-Quan Liang, Tong Wang, Wei-Ou Li, Hui-Juan Li, Sai-Nan Zhu, Dong-Xin Wang

**Affiliations:** 1 Department of Anesthesiology and Critical Care Medicine, Peking University First Hospital, No.8 Xishiku Street, Xicheng District, Beijing, 100034 China; 2 Department of Anesthesiology, Aerospace Central Hospital, No.15 Yuquan Street, Haidian District, Beijing, 100049 China; 3 Peking University Clinical Research Institute, No.38 Xueyuan Road, Haidian District, Beijing, 100191 China; 4 Department of Biostatistics, Peking University First Hospital, No.8 Xishiku Street, Xicheng District, Beijing, 100034 China

**Keywords:** Postoperative complications, Cholinergic antagonists, Penehyclidine, Administration, inhalation, Pre-exposure prophylaxis

## Abstract

**Background:**

Postoperative pulmonary complications (PPCs) are major causes of morbidity, mortality, and prolonged hospital stay in patients after surgery. Using effective strategies to prevent its occurrence is essential to improve outcome. However, despite various efforts, the incidence of PPCs remains elevated in high-risk patients. Anticholinergic inhalation is used to reduce high airway resistance and improve pulmonary function; it may be helpful to decrease the risk of PPCs. Penehyclidine is a long-acting anticholinergic agent which selectively blocks M1 and M3 receptors. We hypothesize that, in high-risk patients, prophylactic penehyclidine inhalation may decrease the incidence of PPCs.

**Methods:**

This is a randomized, double-blind, placebo-controlled trial with two parallel arms. A total of 864 patients at high risk of PPCs will be enrolled and randomized to receive prophylactic inhalation of either penehyclidine or placebo (water for injection). Study drug inhalation will be administered from the night (7 pm) before surgery until the second day after surgery, in an interval of every 12 hours. The primary outcome is the incidence of PPCs within 30 days after surgery. Secondary outcomes include the time to onset of PPCs (from end of surgery to first diagnosis of PPCs), the number of PPCs (indicates the number of diagnosed individual PPCs), the incidence of postoperative extrapulmonary complications, the length of stay in hospital after surgery, and the 30-day all-cause mortality.

**Discussion:**

Results of the present study will provide evidence to guide clinical practice in using prophylactic inhalation of an anticholinergic to prevent PPCs in high-risk patients.

**Trial registration:**

The study was registered prospectively in Chinese Clinical Trial Registry (www.chictr.org.cn, ChiCTR-IPC-15006603) on 14 May 2015 and retrospectively in ClinicalTrials.gov (NCT02644876) on 30 December 2015.

**Electronic supplementary material:**

The online version of this article (doi:10.1186/s13063-017-2315-7) contains supplementary material, which is available to authorized users.

## Background

Postoperative pulmonary complications (PPCs) are major causes of morbidity, mortality, and prolonged hospital stay in patients after surgery [1, 2]. The reported incidences of PPCs vary from 5% to 70% depending on the definition of PPCs, the type of surgical procedures, and the population of patients included in the study [1]. The ARISCAT study identified seven independent predictors, including four patient-related factors and three surgery-related factors, and built a predictive model for the risk of PPCs [3]. A subsequent study confirmed that, in high-risk patients (i.e., patients with cumulative ARISCAT risk score ≥ 45), the incidence of PPCs was as high as 38% (25–51%) [4].

Changes in pulmonary physiology after major thoracic or upper abdominal surgery include diaphragmatic dysfunction, reduced vital capacity, postoperative pain and splinting, and impaired clearance of airway secretions [5]. Affected patients are at increased risk to develop atelectasis, pneumonia, and other respiratory complications. Prophylactic inhalation of anticholinergic agents may be helpful in preventing PPCs by dilating bronchus, decreasing mucous secretion and relieving airway hyperresponsiveness (AHR). In a retrospective study of patients with COPD requiring lung cancer surgery, perioperative inhalation of tiotropium was associated with decreased cardiopulmonary complications after surgery [6]. In a randomized controlled trial of gastric cancer patients with COPD, perioperative (1 week before and 2 weeks after surgery) inhalation of tiotropium decreased the incidence of postoperative complications in those with moderate COPD [7].

Theoretically, selective M1 and M3 receptors blockers may have advantages over nonselective blockers (such as tiotropium bromide) for the purpose of bronchodilation [8]. Penehyclidine is a long-acting anticholinergic agent which selectively blocks M1 and M3 receptors [9, 10]. In a pilot randomized controlled trial of our group, 90 elderly (≥65 years) patients who were admitted to the ICU after long-duration (≥3 hours) surgery randomly received inhalation of penehyclidine, ipratropium bromide, or normal saline for 3 consecutive days. The results showed that prophylactic anticholinergic inhalation significantly reduced the incidence of postoperative bronchospasm (1/30 [3.3%] with penehyclidine hydrochloride, 1/31 [3.2%] with ipratropium bromide, and 6/29 [20.7%] with normal saline, respectively, *P* = 0.025) [11]. We hypothesize that, in high-risk patients, prophylactic inhalation of penehyclidine may decrease the incidence of PPCs.

The purpose of this study is to investigate the impact of prophylactic penehyclidine inhalation on the incidence of PPCs in high-risk patients after major surgery.

## Methods/Design

### Study design

**Fig. 1:**
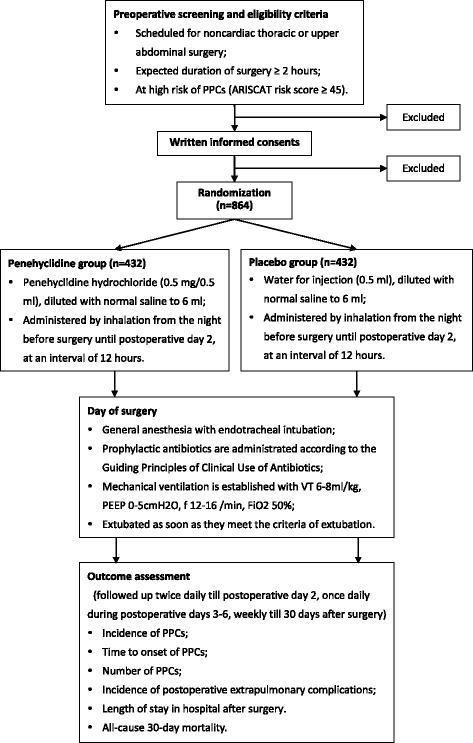
Flowchart of the study

**Fig. 2:**
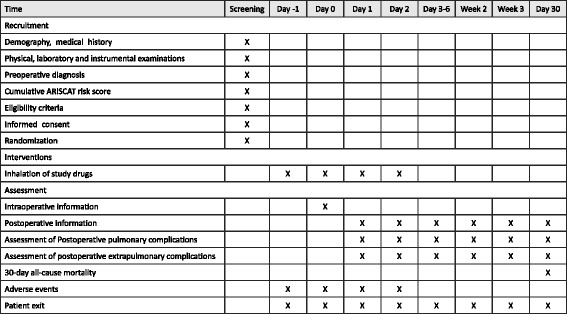
SPIRIT diagram

This registered prospective, single-center, randomized, double-blind, placebo-controlled trial with two parallel arms is to test the superiority of the intervention. Eligible patients will be enrolled and randomly assigned to receive one of two interventions, i.e., penehyclidine hydrochloride inhalation or placebo (water for injection) inhalation (Fig. 1). The study will be coordinated and conducted in the Department of Anesthesiology and Critical Care Medicine of Peking University First Hospital, a tertiary academic hospital in Beijing. The protocol is reported according to the Standard Protocol Items: recommendations for randomized controlled trials (Fig. 2 SPIRIT diagram and Additional file 1 SPIRIT checklist).

### Participants

The inclusion criteria are (1) patients of 50 years or over, (2) scheduled to undergo upper abdominal or noncardiac thoracic surgery with expected duration of 2 hours or longer. For those who undergo thoracoscopic or laparoscopic surgery, the expected length of incision must be 5 cm or more, and (3) judged to be at high risk of PPCs according to the ARISCAT risk score (cumulative ARISCAT risk score ≥ 45, see Table 1).

**Table 1:** ARISCAT (Canet) risk index

Independent predictors of risk for PPCs		Risk score
Age, years		
≤ 50		0
51–80		3
> 80		16
Preoperative SpO_2_, %		
≥ 96%		0
91–95%		8
≤ 90%		24
Respiratory infection in the last month		
No		0
Yes		17
Preoperative anemia (HbO_2_ ≤ 10 g/dl)		
No		0
Yes		11
Surgical incision		
Peripheral		0
Upper abdominal		15
Intrathoracic		24
Duration of surgery, hours		
≤ 2		0
2–3		16
> 3		23
Emergency procedure		
No		0
Yes		8
Risk class	Number of points in risk score	Pulmonary complications rates
Low risk	<26 points	1.6%
Intermediate risk	26–44 points	13.3%
High risk	≥45 points	42.1%

*PPC* postoperative pulmonary complications

Patients who meet any of the following criteria will be excluded: (1) American Society of Anesthesiologists (ASA) physical classification ≥ IV or the expected survival duration ≤ 24 hours; (2) preoperative history of moderate-to-severe symptomatic prostatic hypertrophy or narrow-angle glaucoma; (3) history of myocardial infarction, severe heart dysfunction (New York Heart Association functional classification ≥ 3) or tachyarrhythmia within 1 year; (4) inhalation of β2-receptor activator, M-receptor blockers and/or glucocorticoids within 1 month before surgery; (5) severe renal dysfunction (requirement of renal replacement therapy) or severe hepatic dysfunction (Child-Pugh grade C); (6) history of acute stroke within 3 months before surgery; (7) unable to cooperate in the inhalational therapy; (8) participation in other clinical trial during the last month or within the six half-life periods of the study drug used in the last trial; or (9) refuse to participate in the study.

Criteria of dropout include the following: (1) consent withdrawn by the participants themselves (at least one dose of study drug has been administered); (2) loss to follow‐up; (3) ordered to exit by the investigators or attending physicians (poor compliance, occurrence of severe complications, or occurrence of severe adverse events); (4) prophylactic use of any prohibited drugs (anticholinergics or any medications other than the study drugs) from study recruitment to postoperative day 2; or (5) blindness unmasked. For dropout cases, detailed reasons will be recorded in the case report forms (CRFs). The primary therapeutic effects recorded the last time will be regarded as the final results. These cases will be included in the intention-to-treat analysis but excluded from the per protocol analysis.

Criteria of rejection include the following: (1) consent withdrawn by the participants themselves (study drug is not administered); (2) no study record; or (3) surgery delayed or canceled, unable to assess the occurrence of PPCs. For rejected cases, detailed reasons will be recorded in the CRFs. These cases will be excluded from analysis of therapeutic effects.

### Patient recruitment and baseline data collection

The day before surgery (or Friday for those who will undergo surgery next Monday), investigators who are authorized by the principal investigator will visit patients who are scheduled for upper abdominal or noncardiac thoracic surgery to identify potential participants according to the inclusion and exclusion criteria. They will then invite the potential participants for study participation.

For patients who meet the inclusion/exclusion criteria and provide written informed consent, baseline data including demographic data, preoperative diagnosis, medical history, preoperative medication, surgical history, as well as main results of physical, laboratory, and instrumental examinations will be collected.

### Randomized, blinding and study drug administration

Random numbers were generated in a 1:1 ratio using the SAS 9.2 software package (SAS Institute, Cary, NC, USA) by a biostatistician. Randomization will be stratified according to the type of surgery (upper abdominal or noncardiac thoracic surgery). Study drugs (penehyclidine hydrochloride 1 mg/1 ml, or water for injection 1 ml) are provided as clear aqueous solution in the same 1 ml ampoules (manufactured by Chengdu List Pharmaceutical Co., Ltd., Sichuan, China) and labeled according to the randomization results by a biostatistician and a pharmacist who are independent of patient recruitment and data collection. The results of randomization are sealed in the envelopes and stored at the site of investigation until the end of the study.

During the study period, consecutively recruited patients will be assigned sequential numbers according to which the study drugs will be dispensed by a study coordinator. Study drugs are administered by inhalation in the night (7 pm) before surgery, in the morning (7 am) of surgery, and then every 12 hours until postoperative day 2, resulting in a total number of 7 inhalations. Study drug inhalation is performed with a jet nebulizer (DNA100, manufactured by Congren Medical Equipment Co., Ltd., Xiamen, China) for extubated patients or a vibrating mash nebulizer (Agrogen Professional Nebulizer System, AG-AP6000CH, Aerogen Ltd, Ireland) for patients with mechanical ventilation. Before inhalation, study drugs (penehyclidine hydrochloride 0.5 mg/0.5 ml for patients in the penehyclidine group, or water for injection 0.5 ml for patients in the placebo group) are diluted with normal saline to 6 ml. The performance of each study drug inhalation is recorded in the CRFs.

Patients, investigators, and healthcare team members are all blinded to the study group assignment throughout the study period. In case of an emergency (such as development of a severe adverse event), attending physicians or surgeons can request unmasking of the treatment allocation. These situations will be documented; but analyses will be performed on the intention-to-treat population.

### Anesthesia and perioperative management

Intraoperative monitoring include electrocardiogram, pulse oxygen saturation, noninvasive blood pressure, airway pressure, end-tidal carbon dioxide concentration, nasopharyngeal temperature, urine output, and bispectral index (BIS) value. Intra-arterial pressure (and derivative parameters such as stroke volume variation by FloTrac system) and central venous pressure are monitored when considered necessary.

Anesthetic premedication is not administered. Prophylactic antibiotics (the first or second generation cephalosporin, with or without metronidazole, or cephamycins) are administered 30 minutes to 1 hour before surgical incision. The duration of prophylactic antibiotic treatment is less than 48 hours. The choice of antibiotics and the duration of prophylactic antibiotic therapy are decided according to the *Guiding Principles of Clinical Use of Antibiotics* (published by Chinese National Health and Family Planning Commission in 2015) http://www.nhfpc.gov.cn/zwgkzt/wsbysj/200804/18544.shtml. Therapeutic antibiotic agents are only indicated in patients who are diagnosed with infection.

General anesthesia with endotracheal intubation is performed for all patients. Anesthesia is induced with intravenous propofol or etomidate, sufentanil, and rocuronium, or cisatracurium, and maintained with intravenous propofol, remifentanil and/or sufentanil, and rocuronium or cisatracurium, with or without inhalational sevoflurane and/or nitrous oxide. Neuraxial block is performed depending on the decision of attending anesthesiologists. BIS value is maintained between 40 and 60. Mechanical ventilation is established with a tidal volume from 6 to 8 ml/kg, a positive end-expiratory pressure from 0 to 5 cm H2O, a frequency from 12 to 16 breaths per minute, and 50% oxygen (mixed with nitrous oxide or air). Higher oxygen concentration is administered during one-lung ventilation. Fluid management is performed according to routine practice. Packed red blood cells are transfused to maintain a hemoglobin level above 7 g/dl. Anticholinergics are prohibited unless being used for the treatment of bradycardia, in which case atropine is administered.

At the end of surgery, patients are transferred to the postoperative care unit (PACU) after extubation and monitored for at least 30 minutes before being sent back to the general wards. For patients who are transferred to the intensive care unit (ICU) with endotracheal intubation, propofol and/or dexmedetomidine sedation are provided in addition to opioid analgesia; mechanical ventilation is performed with bilevel ventilation mode (established with a low level at 3 to 5 cm H_2_O, a high level between 13 and 16 cm H_2_O, and a trigger flow at 2 to 3 l/min; PB 840 ventilator, Puritan-Bennett Corp., Mervue, Ireland). Patients are extubated as soon as they meet the criteria of extubation (regain consciousness and airway protective reflex, normal circulatory status and temperature, no residual neuromuscular blockade). For patients requiring mechanical ventilation of more than 24 hours, a ventilator weaning protocol is executed in order to extubate patients as soon as possible (Additional file 2). For patients with acute respiratory failure, noninvasive positive pressure ventilation is used to promote early extubation or to avoid reintubation. For all patients after extubation, low-flow supplemental oxygen is routinely provided during the day of surgery.

During the postoperative period, patient-controlled analgesia (either intravenous or epidural) is provided for up to 3 days after surgery. The target is to maintain a Numeric Pain Rating Scale (NPRS, an 11-point scale where 0 = no pain and 10 = the worst pain) pain score of 3 or less. Nonsteroidal anti-inflammatory drugs are administered when considered necessary and without contraindications. Other treatments including chest physiotherapy, early mobilization, removal of nasogastric tube, parenteral or enteral nutrition, and intravenous respiratory medications (such as expectorants, theoclears and glucocorticoids) are administered according to routine practice.

During the study period, inhalations of any respiratory medications for prophylactic purpose other than the study drugs are prohibited. However, for patients who develop PPCs, inhalation of respiratory medications is allowed. For those who develop harmful effects or clinical deterioration, the dose of study drugs can be decreased or the study drug administration can be discontinued on request of participants, attending surgeons or investigators. These situations will be recorded in the CRFs.

### Patient follow-up and outcome assessment

Patients will be followed up twice daily until postoperative day 2, once daily from postoperative days 3 to 6, and then weekly until 30 days after surgery. Intraoperative data including type and duration of anesthesia, uses and doses of anesthetics, analgesics and other drugs, parameters of mechanical ventilation, fluid balance and transfusion of blood products, as well as type and duration of surgery will be collected. The final cumulative ARISCAT risk score will be calculated just after the surgery. Postoperative data including NRS pain scores, analgesic consumption, respiratory management, return of gastrointestinal function, start of nutrition, occurrence of complications and related treatment, admission to the ICU, duration of hospital stay, and any events that lead to return or readmission to hospital after hospital discharge will be recorded. Any adverse events or complications that occur during the study period will be promptly treated according to routine practice. For patients who deviate from the intervention protocol, deviations will be corrected when possible and follow-up will be completed. For patients who withdraw consent, the last follow-up results will be regarded as the final results.

#### Primary outcome

The primary outcome is the incidence of PPCs within 30 days after surgery. In the present study, PPCs are generally defined as any condition that affects the respiratory tract system and may adversely influence patients’ outcome [1]. The diagnostic criteria of each individual PPC are listed in Table 2 [3]. We adopt the Clavien-Dindo Classification to categorize PPCs into five major groups [12, 13], and PPCs of grade II or above will be used to calculate the incidence of PPCs (Additional file 3). If a PPC occurs during the follow-up period, the date of earliest diagnosis and the evidences according to which the diagnosis is made will be documented.

**Table 2:** Definitions of PPCs

Complications	Definition
Respiratory infections	Receiving antibiotics for a suspected respiratory infection and met at least one of the following criteria: new or changed sputum, new or changed lung opacities, fever, leukocyte count >12 × 10^9^/L
Respiratory failure	PaO_2_ < 60 mmHg on room air, a ratio of PaO_2_ to inspired oxygen fraction < 300, or arterial oxyhemoglobin saturation measured with pulse oximetry < 90% and requiring oxygen therapy
Pleural effusion	Chest X-ray demonstrating blunting of the costophrenic angle, loss of the sharp silhouette of the ipsilateral hemidiaphragm in upright position, evidence of displacement of adjacent anatomical structures, or (in supine position) a hazy opacity in one hemithorax with preserved vascular shadows
Atelectasis	Lung opacification with a shift of the mediastinum, hilum, or hemidiaphragm toward the affected area, and compensatory overinflation in the adjacent nonatelectatic lung
Pneumothorax	Air in the pleural space with no vascular bed surrounding the visceral pleura
Bronchospasm	Newly detected expiratory wheezing treated with bronchodilators
Aspiration pneumonitis	Acute lung injury after inhalation of regurgitated intragastric contents

*PPCs* postoperative pulmonary complications

#### Secondary outcomes

Secondary outcomes include the time to onset of PPCs (from end of surgery to first diagnosis of PPCs), the number of PPCs (indicate the number of diagnosed individual PPCs), the incidence of postoperative extrapulmonary complications, the length of stay (LOS) in hospital after surgery, and the 30-day all-cause mortality. Postoperative extrapulmonary complications are defined as complications other than PPCs that occur within 30 days after surgery and require therapeutic intervention. For each diagnosed extrapulmonary complications, the date of earliest diagnosis and the evidences according to which the diagnosis is made will also be documented.

#### Adverse events

An adverse event indicates any unpredictable, unfavorable medical event that occurs during the period of study drug administration. It can be related to the study intervention or otherwise. Exceptional adverse events occurred during anesthesia include (but not limited to) desaturation (PaO_2_ < 60 mmHg or SpO_2_ < 90%), high airway pressure (peak airway pressure > 40 mmHg), hypercapnia (PaCO_2_ > 50 mmHg), difficult airway (failed intubation attempt > 3 times), hypotension (systolic blood pressure < 90 mmHg or a decrease of more than 30% from baseline), hypertension (systolic blood pressure > 180 mmHg or an increase of more than 30% from baseline), bradycardia (heart rate < 40 beats per minute), tachycardia (heart rate > 100 beats per minute). Other possible adverse events occurred during perioperative period include (but not limited to) nausea, dry month, flushing, dizziness, palpitation, somnolence, cough, delirium, etc. These events are diagnosed according to patient’s complains and symptoms (sought by non-directive questioning), physical and mental signs, as well as results of laboratory tests and instrumental examinations. Delirium is assessed with the confusion assessment method for the intensive care unit (CAM-ICU). Any developed adverse events are managed according to routine practice and followed up until they are properly resolved, stabilized or recovered to normal. The information including the diagnosis, date(s) of onset and resolution (if applicable), severity of influence, relationship with intervention, treatment and outcomes (sequelae) are recorded. The occurrence of adverse events will be reported to the ethics committee in the final report.

A severe adverse event indicates any unpredictable medical events that lead to death, threat of life, prolonged length of hospital stay, persistent disability or dysfunction, or other severe event. All severe adverse events will be monitored, managed, followed up and recorded as above, and will be reported to the ethics committee within 24 hours after their occurrence. Harmful consequence resulted directly from study participation will be compensated according to the corresponding legal provisions.

### Sample size calculation

In our recent randomized controlled trial, PPCs occurred in 51.7% of patients in the placebo group [11]. Mazo et al. reported incidences of PPCs from 42.1% to 44.9% in high-risk patients (ARISCAT risk score ≥ 45) [4]. Whereas perioperative inhalation of tiotropium reduced the incidence of PPCs by 16% [6], and inhalation of penehyclidine during the first three postoperative days reduced the incidence of PPCs by 12.4% [11]. In the present study, we assume that the incidence of PPCs will be 42.1% in high-risk patients of the placebo group, and prophylactic penehyclidine inhalation will reduce the incidence of PPCs by 10%. The calculated sample size that will provide 80% power to detect a superiority of this difference at a one-sided significance level of 0.025 is 360 patients in each group. Considering a dropout rate of about 20%, we plan to enroll 432 patients in each group. The sample size calculation was performed with PASS 2008 software.

### Data management

Investigators are trained to record data promptly, completely, and correctly in the CRFs according to original observation. On-site auditing will be performed at least three times during the study period, with reports and feedbacks provided in written form. Data input will be performed with EpiData software (Version 3.1). Dataset will be locked when the following tasks have been completed: (1) patient recruitment and follow-up are completed, (2) data collection, input and double-checked is performed without errors, and (3) all data queries have been solved. Data management will be performed by Peking University Clinical Research Institute who is independent from the investigators and sponsors.

### Data analysis

#### General principles

All statistical analyses will be performed with SPSS 14.0 software package (SPSS, Chicago, IL, USA) and SAS 9.2 software (SAS Institute, Cary, NC, USA) by statisticians in the Department of Biostatistics of Peking University First Hospital. Since the safety of intervention has been confirmed by our previous pilot study, interim analysis is not planned. Analyses will be performed on an intention-to-treat basis, that all subjects will be analyzed in the group which they were assigned to. For the primary outcome (the incidence of PPCs within 30 days after surgery), per protocol analysis will also be performed.

#### Baseline data

Statistical description will be provided for baseline data such as demographic variables, medical history, preoperative medications, and perioperative management.

#### Outcome analysis

Primary outcome (the incidence of PPCs within 30 days after surgery) will be compared with χ2 test. The difference between groups and the 95% confidence interval of the difference will be calculated.

For secondary outcomes, continuous variables with normal distribution will be analyzed using an unpaired *t* test; continuous variables with abnormal distribution or ranked data will be analyzed using Mann-Whitney *U* test; categorical variables will be analyzed using the chi-squared test, continuity correction chi-squared test or Fisher exact test; time-to-event results will be analyzed using the Kaplan-Meier estimator, and the differences between groups will be tested by the log-rank test. Subgroup analyses will be performed according to the type of the surgery (upper abdominal or thoracic).

## Discussion

This randomized, double-blind, placebo-controlled trial with two-parallel arms is designed to test the hypothesis that prophylactic penehyclidine inhalation can reduce the incidence of PPCs in high-risk patients undergoing major thoracic and upper abdominal surgery.

Identifying patient at high risk of PPCs is the first step of our study in order to guarantee the efficiency of comparison. Various predictive models have been reported to identify patients at high risk of pneumonia, respiratory failure, pulmonary complications, and cardiopulmonary complications after surgery [1]. In the present study, we adopt the ARISCAT risk score to identify high-risk patients [3] because of the following reasons. First, this scoring system is derived from patients undergoing various types of surgery (including thoracic and abdominal surgery) under various type of anesthesia. Therefore, it is suitable for the patient population in our study. Second, the risk indices are convenient to use. Besides the four patient-related factors, the three surgery-related factors can also be known or anticipated before recruitment. Third, the predictive effect of this scoring system has been validated in a recent study [4].

PPCs are a composite of the hospital-acquired respiratory events after surgery. The diagnostic criteria used to define individual PPCs varied among different studies. In the present study, we used the diagnostic criteria used in the ARISCAT study [3]. In addition, we adopt the Clavien-Dindo Classification to categorize PPCs, and those of grade II or above will be used to calculated the incidence of PPCs [12, 13]. For example, patients with a small amount pleural effusion that does not require specified medical management (e.g., diuretics) or intervention (e.g., drain placement) will be excluded from the diagnosis. This will help us to identify PPCs that have impact on the prognosis of patients.

Using effective strategies to prevent PPCs is essential in high-risk patients. Some strategies have been proved beneficial in previous studies, such as smoking cessation [14] and inspiratory muscle training [15, 16] before surgery; use of regional anesthesia [17, 18], lung-protective ventilation (for patients who received general anesthesia) [19, 20] and goal-directed fluid therapy [21] during surgery; as well as use of continuous positive airway pressure or noninvasive ventilation [22, 23] and early exercise [24, 25] after surgery. High-risk patients, such as those complicated with bronchial asthma, chronic obstructive pulmonary disease (COPD), pneumonia, left ventricular dysfunction and morbid obesity, tend to develop AHR during the perioperative period [26–28], which increase the risk of PPCs. Whereas anticholinergic inhalation can prevent parasympathetic bronchoconstrictive effect and reduce high airway resistance [29, 30], and improve patients’ pulmonary function [31, 32]. As a long-acting anticholinergic drug with selective M1, M3-receptors blocking activity [10], penehyclidine is a promising candidate agent in the treatment of COPD [33]. Indeed, penehyclidine inhalation was used in COPD patients with acute exacerbation and resulted in good effectiveness [34]. Our pilot study suggested that penehyclidine inhalation might be effective in preventing PPCs in high-risk patients [11]. Results of the present study will provide evidence on the effectiveness and safety of penehyclidine inhalation in preventing PPCs in high-risk surgical patients.

Our study has several limitations. First, unlike other inhalational anticholinergics (such as tiotropium bromide and ipratropium bromide), penehyclidine is currently only approved for clinical use in China. This will restrict the generalizability and reproducibility of our results. However, considering the selective M1 and M3 receptor blocking activity and long-lasting effects, penehyclidine may be more suitable for PPCs prevention. Second, some perioperative factors, such as selection of anesthetic methods, use of anesthetics and analgesics, fluid management, respiratory management and ventilator settings, may also affect the occurrence of PPCs. We do not standardize anesthetic management in the present study. This might produce confounding effects to our intervention. However, large sample size and strict randomization will help to balance these factors between groups. And our results will reflect the impact of penehyclidine inhalation on PPC incidence in the real-world practice.

## Trial status

Recruitment and data collection of patients started on 1 September 2015. The recruitment of 864 patients at high-risk of PPCs is scheduled to end on 1 August 2018. Data analysis and evaluation will be performed subsequently. The final results of this study will be published.

## Additional files

Additional file 1: SPIRIT checklist. (DOC 130 kb)

Additional file 2: Ventilator weaning protocol. (DOCX 29 kb)

Additional file 3: Criteria of grade of PPCs according to the Clavien-Dindo classification. (DOCX 18 kb)

Additional file 4: WHO Trial Registration Dataset. (DOC 49 kb)

## Abbreviations

AHR: Airway hyperresponsiveness
ASA: American Society of Anesthesiologists
CAM-ICU: Confusion assessment method for the intensive care unit
COPD: Chronic obstructive pulmonary disease
CRFs: Case report forms
ICU: Intense care unit
NPRS: Numeric Pain Rating Scale
PACU: Post-anesthesia care unit
PPCs: Postoperative pulmonary complications
