# Supplementary material for: Prophylactic penehyclidine inhalation for prevention of postoperative pulmonary complications in high-risk patients: study protocol of a randomized controlled trial
Source: Trials. 2017 Nov 28;18:571. doi: 10.1186/s13063-017-2315-7 (PMC5706155; doi:10.1186/s13063-017-2315-7)
Supplement: Supplementary file 2 — Ventilator weaning protocol. (DOCX 29 kb) [file 13063_2017_2315_MOESM2_ESM.docx]

Patients requiring mechanical ventilation ≥ 24h

Monitor weaning readiness criteria daily:

- Evidence of reversal of the underlying cause for respiratory failure;
- Adequate oxygenation (eg, PaO_2_/FiO_2_ > 150-200 mmHg) on low PEEP (≥ 5-8 cm H_2_O) and pH ≥ 7.25;
- Hemodynamic stability: no active myocardial ischemic, no clinically important hypotension;
- The patient is able to initiate an inspiratory effort.

**SBT (Spontaneous breathing trail)**

- Usually 30 minutes to two hours;
- Through a T-piece or with minimal ventilator support (PSV 5-8 cm H_2_O).

**Assessment of airway protection and airway patency**

- White card test;
- Cuff leak test.

Patient meets criteria?

Pass?

Pass?

**Extubate**

Increase support or return to full support

No

No

No

Yes

Yes

Yes

**References:**

Haas CF, Loik PS. Ventilator Discontinuation Protocols. [Respir Care.](https://www.ncbi.nlm.nih.gov/pubmed/23013902) 2012;57(10):1649-62.
