# Supplementary material for: Prophylactic penehyclidine inhalation for prevention of postoperative pulmonary complications in high-risk patients: study protocol of a randomized controlled trial
Source: Trials. 2017 Nov 28;18:571. doi: 10.1186/s13063-017-2315-7 (PMC5706155; doi:10.1186/s13063-017-2315-7)
Supplement: Supplementary file 3 — Criteria of grade of PPCs according to the Clavien-Dindo classification. (DOCX 18 kb) [file 13063_2017_2315_MOESM3_ESM.docx]

**Additional file 3. Criteria of grade of PPCs according to the Clavien-Dindo classification**

| Complications | I | II | IIIa | IIIb | IVa | IVb | V |
| --- | --- | --- | --- | --- | --- | --- | --- |
| Respiratory infections | Clinical observation or diagnostic evaluation only; intervention not indicated except for nebulizers, expectorants, or lung physiotherapy (e.g., postural drainage) | Medical management indicated (e.g., antibiotics) | Intervention not under general anesthesia (e.g., bronchoscopic aspiration, tracheal puncture) | Intervention under general anesthesia (e.g., tracheostomy under general anesthesia or sedation) | Mechanical ventilation indicated | Sepsis or multiple organ failure | Death |
| Respiratory failure | _ | _ | _ | _ | Mechanical ventilation indicated | Sepsis or multiple organ failure | Death |
| Pleural effusion | Clinical observation or diagnostic evaluation only; intervention not indicated (drainage only through existing drainage tube) | Medical management indicated (e.g., diuretics) | Intervention not under general anesthesia (e.g., Image-guided drain placement or thoracentesis including drain replacement indicated) | Intervention under general anesthesia indicated | Mechanical ventilation indicated | Multiple organ failure | Death |
| Atelectasis | Clinical observation or diagnostic evaluation only; intervention not indicated, except for nebulizers, expectorants, or lung physiotherapy (e.g., postural drainage) | Medical management indicated (e.g., antibiotics) | Intervention not under general anesthesia (e.g., bronchoscopic aspiration, tracheal puncture) | Intervention under general anesthesia (e.g., tracheostomy under general anesthesia or sedation) | Mechanical ventilation indicated | Sepsis or multiple organ failure | Death |
| Pneumothorax | Clinical observation or diagnostic evaluation only; intervention not indicated (drainage only through existing drainage tube) | _ | Intervention not under general anesthesia (e.g., closed drainage of thoracic cavity or thoracentesis including drain replacement indicated) | Intervention under general anesthesia indicated | Mechanical ventilation indicated | Multiple organ failure | Death |
| Bronchospasm | Clinical observation or diagnostic evaluation only; intervention not indicated except for nebulizers (bronchodilators not included), expectorants, or lung physiotherapy (e.g., postural drainage) | Medical management indicated (e.g., bronchodilators) | _ | _ | Mechanical ventilation indicated | Multiple organ failure | Death |
| Aspiration pneumonitis | Clinical observation or diagnostic evaluation only; intervention not indicated except for nebulizers, expectorants, or lung physiotherapy (e.g., postural drainage) | Medical management indicated (e.g., antibiotics, or bronchodilators, or glucocorticoids) | Intervention not under general anesthesia (e.g., bronchoscopic aspiration) | Intervention under general anesthesia (e.g., tracheostomy under general anesthesia or sedation) | Mechanical ventilation indicated | Sepsis or multiple organ failure | Death |

**References**

1. [Dindo D](https://www-ncbi-nlm-nih-gov.libproxy.ucl.ac.uk/pubmed/?term=Dindo%20D%5BAuthor%5D&cauthor=true&cauthor_uid=15273542), [Demartines N](https://www-ncbi-nlm-nih-gov.libproxy.ucl.ac.uk/pubmed/?term=Demartines%20N%5BAuthor%5D&cauthor=true&cauthor_uid=15273542), [Clavien PA](https://www-ncbi-nlm-nih-gov.libproxy.ucl.ac.uk/pubmed/?term=Clavien%20PA%5BAuthor%5D&cauthor=true&cauthor_uid=15273542). Classification of surgical complications: a new proposal with evaluation in a cohort of 6336 patients and results of a survey. Ann Surg. 2004; 240:205-13.
2. [Katayama H](https://www-ncbi-nlm-nih-gov.libproxy.ucl.ac.uk/pubmed/?term=Katayama%20H%5BAuthor%5D&cauthor=true&cauthor_uid=26289837), [Kurokawa Y](https://www-ncbi-nlm-nih-gov.libproxy.ucl.ac.uk/pubmed/?term=Kurokawa%20Y%5BAuthor%5D&cauthor=true&cauthor_uid=26289837), [Nakamura K](https://www-ncbi-nlm-nih-gov.libproxy.ucl.ac.uk/pubmed/?term=Nakamura%20K%5BAuthor%5D&cauthor=true&cauthor_uid=26289837), [Ito H](https://www-ncbi-nlm-nih-gov.libproxy.ucl.ac.uk/pubmed/?term=Ito%20H%5BAuthor%5D&cauthor=true&cauthor_uid=26289837), [Kanemitsu Y](https://www-ncbi-nlm-nih-gov.libproxy.ucl.ac.uk/pubmed/?term=Kanemitsu%20Y%5BAuthor%5D&cauthor=true&cauthor_uid=26289837), [Masuda N](https://www-ncbi-nlm-nih-gov.libproxy.ucl.ac.uk/pubmed/?term=Masuda%20N%5BAuthor%5D&cauthor=true&cauthor_uid=26289837), et al. Extended Clavien-Dindo classification of surgical complications: Japan Clinical Oncology Group postoperative complications criteria. Surg Today. 2016;46:668-85.
