# Supplementary material for: Prophylactic penehyclidine inhalation for prevention of postoperative pulmonary complications in high-risk patients: study protocol of a randomized controlled trial
Source: Trials. 2017 Nov 28;18:571. doi: 10.1186/s13063-017-2315-7 (PMC5706155; doi:10.1186/s13063-017-2315-7)
Supplement: Supplementary file 4 — WHO Trial Registration Dataset. (DOC 49 kb) [file 13063_2017_2315_MOESM4_ESM.doc]

**Additional file 4. WHO Trial Registration Data Set**

| **Data category** | **Information** |
| --- | --- |
| Primary registry and trial identifying number | Chinese Clinical Trial Registry ChiCTR-IPC-15006603; |
| Date of registration in primary registry | Chinese Clinical Trial Registry ([www.chictr.org.cn](http://www.chictr.org.cn/), ChiCTR-IPC-15006603) on May 14^th^, 2015 |
| Secondary identifying numbers | ClinicalTrials.gov NCT02644876 |
| Source(s) of monetary or material support | Chinese Society of Cardiothoracic and Vascular Anesthesiology  Peking University First Hospital  Chengdu List Pharmaceutical Co, Ltd, Sichuan, China |
| Primary sponsor | Peking University First Hospital |
| Secondary sponsor(s) |  |
| Contact for public queries | Dong-Xin Wang, MD, PhD, E-mail: [wangdongxin@hotmail.com](mailto:wangdongxin@hotmail.com) |
| Contact for scientific queries | Dong-Xin Wang, MD, PhD  Department of Anesthesiology and Critical Care Medicine  Peking University First Hospital  No.8 Xishiku Street, Xicheng District  Beijing 100034, China  E-mail: [wangdongxin@hotmail.com](mailto:wangdongxin@hotmail.com) |
| Public title | Prophylactic penehyclidine inhalation for prevention of postoperative pulmonary complications in high-risk patients |
| Scientific title | Prophylactic penehyclidine inhalation for prevention of postoperative pulmonary complications in high-risk patients: a randomized controlled trial |
| Countries of recruitment | China |
| Health condition(s) or problem(s) studied | Postoperative Pulmonary Complications; Cholinergic Antagonists; Penehyclidine; Inhalation |
| Intervention(s) | Experimental: penehyclidine hydrochloride will be administered by inhalation (penehyclidine hydrochloride 0.5mg/0.5ml+normal saline 5.5ml) once every 12 hours from the night before surgery till postoperative day 2, resulting a total number of 7 inhalations) |
|  | Placebo comparator: Placebo will be administered by inhalation (water for injection 0.5ml+normal saline 5.5ml) once every 12 hours from the night before surgery till postoperative day 2, resulting a total number of 7 inhalations) |
| Key inclusion and exclusion criteria | The inclusion criteria are (1) patients of 50 years or over, (2) scheduled to undergo upper-abdominal or noncardiac thoracic surgery with expected duration of 2 hours or longer. For those who undergo thoracoscopic or laparoscopic surgery, the expected length of incision must be 5 centimeters or more, and (3) judged to be at high risk of PPCs according to the ARISCAT risk score. |
|  | The exclusion criteria are (1) ASA classification ≥ IV or the expected survival duration ≤ 24 h; (2) preoperative history of moderate-to-severe symptomatic prostatic hypertrophy or narrow-angle glaucoma; (3) history of myocardial infarction, severe heart dysfunction or tachyarrhythmia within one year; (4) inhalation of β2-receptor activator, M-receptor blockers and/or glucocorticoids within one month before surgery; (5) severe renal dysfunction or severe hepatic dysfunction; (6) history of acute stroke within three months before surgery; (7) unable to cooperate with the inhalational therapy; (8) participation in other clinical trial during the last month or within the six half-life periods of the study drug used in the last trial; or (9) refuse to participate in the study. |
| Study type | Interventional |
|  | Allocation: randomized; Intervention model: parallel assignment; Masking: double blind |
|  | Primary purpose: prevention |
|  | Phase IV |
| Date of first enrolment | September 1^st^, 2015 |
| Target sample size | 864 |
| Recruitment status | Recruiting |
| Primary outcome(s) | The incidence of PPCs within 30 days after surgery |
| Key secondary outcomes | The time to onset of PPCs, the number of PPCs, the incidence of postoperative extrapulmonary complications, the length of stay (LOS) in hospital after surgery, and the 30-day all-cause mortality. |
